# Cocaine-Induced Cardiac Alterations: Histological and Immunohistochemical Post-Mortem Analysis

**DOI:** 10.3390/diagnostics15080999

**Published:** 2025-04-14

**Authors:** Valeria Palumbo, Michele Treglia, Manuel Scimeca, Francesca Servadei, Erica Giacobbi, Rita Bonfiglio, Margherita Pallocci, Pierluigi Passalacqua, Fabio Del Duca, Roberta Tittarelli, Luca Coppeta, Stefania Schiaroli, Giulio Cervelli, Alessandro Mauriello, Luigi Tonino Marsella, Silvestro Mauriello

**Affiliations:** 1 Department of Experimental Medicine, Tor Vergata Oncoscience Research, University of Rome “Tor Vergata”, 00133 Rome, Italyfrancescaservadei@gmail.com (F.S.);; 2 Department of Biomedicine and Prevention, University of Rome “Tor Vergata”, 00133 Rome, Italy; 3 PhD School of Applied Medical-Surgical Sciences, University of Rome “Tor Vergata”, 00133 Rome, Italy; 4 Department of Public Health and Infectious Diseases, “Sapienza” University of Rome, 00185 Rome, Italy; 5 Department of Anatomical, Histological, Forensic and Orthopedic Sciences, Sapienza University of Rome, 00185 Rome, Italy

**Keywords:** cocaine, cardiac alterations, legal medicine, inflammatory biomarkers, forensic toxicology, immunohistochemistry, histological alterations

## Abstract

**Background:** Cocaine abuse represents a serious health issue. The cardiovascular system is one of the main sites on which cocaine elicits its toxicity, as indicated by deadly events mainly related to myocardial infarction. The main aim of this study was to characterize the histological and immunohistochemical alterations related to cocaine abuse in cardiac tissue. **Methods:** Cardiac tissue samples derived from cocaine-related (*n* = 30) and not-cocaine-related deaths (*n* = 30). Histomorphology evaluations and immunohistochemistry for inflammatory biomarkers (CD45 and CD3) have been performed on formalin-fixed, paraffin-embedded (FFPE) cardiac tissue samples. **Results:** A higher frequency of cardiac alterations, such as wavy fibers, interstitial edema, fibrosis and hemorrhagic extravasation, were found in the group of cocaine users compared to the control group. Moreover, immunohistochemical analysis showed higher levels of inflammatory cells infiltrate within the cocaine-related deaths group. **Conclusions:** These data could shed new light on the complex relationship between cocaine use and cardiac alterations. Specifically, our data support the evidence that cocaine abuse is related to cardiac inflammation. Therefore, the generation of an inflammatory state could promote functional and structural cardiac alterations and lead ultimately to myocardial infarction. This would explain the high frequency of acute myocardial infarction in cocaine users.

## 1. Introduction

Cocaine is one of the most consumed drugs, with the highest prevalence levels found in North and South America and Central/Western Europe [1]. Cocaine addiction and its associated health consequences, including cocaine-related deaths, remain a significant concern on a global scale. Recent data on cocaine abuse are alarming: according to the 2020 UNODC report, an estimated 21.5 million people have used cocaine at least once, representing 0.4 percent of the global population aged 15–64 [2]. Cocaine abuse is frequent in men, which represent 73% of the cocaine-addicted population.

There is a set of negative health consequences related to cocaine use due to its high potential for abuse and addiction. Cocaine-related health impairment depends on the type of cocaine product (e.g., powdered hydrochloride salt or “crack”) as well as the administration route (e.g., smoking, intravenous injection, nasal inhalation, or oral application), but in general, it is related to both physical and mental disorders, such as mood and sleep disorders, paranoia, suspiciousness, schizophrenia, depression and suicidal and homicidal thinking [3].

Cocaine initially exerts its effects almost immediately after administration (1–5 min), and its toxicity is demonstrated by an increased heart rate and blood pressure, and vasoconstriction leading to the blocking of sodium/potassium channels and resulting in an increased risk of myocardial infarction [2,4].

Concerning cardiovascular failure, cocaine can trigger potentially fatal adverse cardiovascular events, and its cardiotoxic effects have a multifactorial origin [5,6]. Cocaine could be related to acute or chronic damage by both direct and indirect mechanisms.

Regarding acute toxicity, this can be related to a single as well as a limited number of drug administrations, or to a “binge use”. To differentiate between acute or chronic toxicity can be challenging, given that it is arduous to define blood concentrations of cocaine that are fatal and that, conversely, even relatively low levels of cocaine in the blood can cause myocardial infarction related to coronary artery spasm, especially in individuals with underlying heart diseases.

Moreover, the concurrent use of cocaine with other psychotropic substances (e.g., alcohol and/or heroin) significantly raises cocaine levels in the blood (up to 30%), causing increased, prolonged cardiovascular risks. The cardiovascular risk is particularly elevated mainly in the cases of concomitant use of alcohol and cocaine, because of the production of cocaethylene, which may potentiate the cardiotoxic effects of both substances [7]. Co-use of cocaine and cannabis has also been highlighted as a situation that increases the risk of adverse cardiological events [8].

Some extreme situations of accidental administration of cocaine in supralethal doses are seen in “drug traffickers/packers” (carrying considerable amounts of the substance by hiding it within their body cavities) and “drug swallowers”, typically those involved in selling the drug who, frightened of being caught in the event of a police raid, swallow it. In these cases, the rupture of cocaine packages instantly releases a supralethal dose that causes very severe symptoms of cardiac standstill, in addition to seizures, intracranial bleeding, bowel ischemia, respiratory depression and even death [9].

The acute toxicity of cocaine is due to mechanisms leading to hypertension, arrhythmia, coronary artery aneurysms (CAAs) and acute myocardial infarction (MI) [10,11]. It has been shown that cocaine use is also correlated to transitory ischemic attacks (TIAs) and silent cerebrovascular accidents (CVAs) [12]. Other effects seen in acute intoxication include behavioral activation, impulsivity and acute behavioral disorders, which may lead to traumatic accidents, violence or self-harm episodes [13,14].

The major acute toxicities induced by cocaine result from its sympathomimetic effects [15]. The sympathomimetic stimulation of cocaine abuse on the heart and vascular smooth muscle leads to increased norepinephrine levels in vascular smooth muscle. This, in turn, stimulates postsynaptic α receptors, resulting in increased calcium flow, a vasoconstrictive response, and subsequently, elevated blood pressure and coronary vascular resistances [16,17].

This effect on the vascular smooth muscle can be considered as the cause of the cocaine-induced vascular ischemia that can, at least in part, explain the reported larger infarctions of other organs like the kidney and spleen, including acute myoglobinuric renal failure. Ischemic events which are cocaine-related have also been shown to involve the skin, the intestine and the aorta with aortic dissection [12].

Alternatively, the vasoconstrictive response of cocaine could be traced to a direct stimulus to calcium flow within smooth muscle cells. Moreover, the accumulation of catecholamines in the myocardium stimulates β-adrenergic receptors, resulting in increased cardiac automaticity and thus the onset of tachyarrhythmias [18,19]. The stimulation of β-adrenergic receptors in the heart results in increased levels of free calcium in myocardial cells, leading to a positive inotropic effect. This effect is reported for low doses of cocaine and could contribute to the formation of contraction bands associated with MI [19,20]. These phenomena are part of the pathogenesis of MI, a common event related to cocaine acute toxicity [21]. The blockade of norepinephrine reuptake from sympathetic nerve endings in the cardiovascular system can significantly increase heart rate, blood pressure and systemic vascular resistances. Likewise, through the stimulation of α- and β-adrenergic receptors, cocaine increases the calcium concentration in cardiomyocytes, thereby facilitating myocardial contractility by calcium uptake. Long-term cocaine abuse could also lead to chronic toxic effects on the cardiovascular system. Chronic cocaine use causes irreversible structural damage to the heart, including cardiomyopathies such as left ventricular hypertrophy, but also arterial endothelium and vascular damage, and the progression of atherosclerosis [22,23]. Since the early evidence, cocaine has been shown to result in sudden death in 76% of cases in individuals with an initially low risk of atherosclerosis [24]. It seems that cocaine is capable of accelerating atherosclerosis of coronary arteries. The mechanism involves thrombus formation due to increased coronary vasoconstriction and increased platelet aggregation and thromboxane production [25,26,27]. In addition, cocaine-induced coronary vasospasm can severely damage the coronary endothelium and cause adhesion, aggregation and thrombus formation [28]. In this regard, a study conducted in an emergency department setting has demonstrated endothelial dysfunction in patients with acute cocaine overdose. Specifically, it has been shown that an elevation in serum siCAM-1 (soluble intercellular adhesion molecule-1) has been associated with altered vasoreactivity, blunted vasodilatation and chronic cocaine use [29].

From a diagnostic point of view, it has been highlighted that in the clinical context, cardiac magnetic resonance imaging is one of the most promising methods to detect myocardial dysfunction in cocaine users, both acute and chronic, and even if it is clinically asymptomatic [30].

Although some information about cocaine’s toxicological effects on the cardiovascular apparatus have been described in the literature, at the state of the art, few histological post-mortem studies are available. Histological features of the heart determined by cocaine toxicity, especially in humans, must be better identified, to promote a deeper insight into the underlying pathophysiological mechanisms and to provide a useful diagnostic tool for the forensic pathologist in cases of cocaine-related death.

Starting from these considerations, the main aim of this study was to better characterize the histopathological alterations related to cocaine abuse in cardiac tissue samples. In addition, this study aims to investigate the possible effects of cocaine abuse on the intracardiac inflammatory infiltrate.

## 2. Materials and Methods

### 2.1. Case Selection

In the present study, heart samples from a population of 60 cadavers have been collected to evaluate the occurrence of cocaine-induced cardiac alterations. Among them, 30 samples have been collected from a control group consisting of 27 males and 3 females negative for the presence of cocaine in the blood, deceased due to head trauma, primarily resulting from a traffic accident, and a positive group of 30 samples from 26 males and 4 females whose death was instead related to cocaine use. Toxicological examinations were conducted in femoral blood samples of all cadavers. Cases were excluded if there was a histological diagnosis of myocardial infarction, myocarditis and coronary artery disease. Decomposed bodies or those with initial signs of putrefaction were excluded from both groups. Autoptic investigations were performed within 36 h after death.

In all selected cases, there was no history of cardiac pathologies. The mean age of the case group was 33.3 years (range 19–57). The mean age of the control group was 35.4 years.

### 2.2. Autopsy Procedure and Tissue Processing

The autopsies were performed in accordance with the European guidelines on medicolegal autopsy. Hearts were evaluated according to the guidelines for autopsy investigation of SCD: 2017 update from the Association for European Cardiovascular Pathology. Specifically, the weight, wall thickness and transverse and longitudinal diameters were recorded for each heart.

The following heart samples were collected. Left ventricle: anterior wall, lateral wall, posterior wall, posterior septum, anterior septum and apex. Right ventricle: posterior wall.

**Figure 1:**
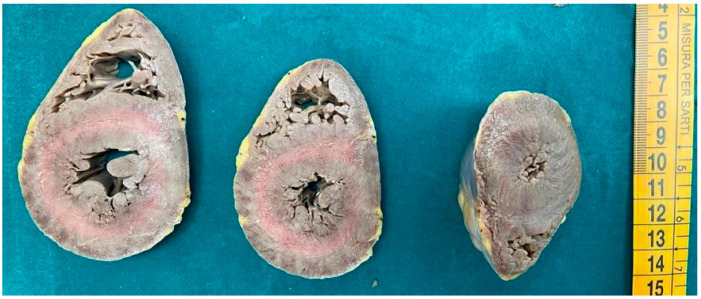
Heart of a subject who died from cocaine abuse. The organ was sectioned, after fixation in formaldehyde, by means of three longitudinal sections. A thickening of the parts and a reduction of the ventricular chambers, as in hypertrophic cardiomyopathy, can be observed.

Myocardial and coronary artery sections were obtained and processed for standard histological examination (Figure 1). Tissue samples were fixed in 4% buffered formalin and paraffin embedded [31]. Four µm-thick sections were stained with hematoxylin and eosin (HE), Masson’s trichrome stain, Sirius’ red stain and Van Gieson stain.

### 2.3. Immunohistochemical Analysis

Immunohistochemical analyses were performed to investigate the presence of CD45 and CD3 lymphocytes in all collected samples. Specifically, serial sections were pre-treated using heat-mediated antigen retrieval with EDTA citrate (pH 7.8) for 30 min at 95 °C [32,33,34]. The section was then incubated with the primary monoclonal antibodies anti-CD45 (mouse monoclonal clone 2B11 & PD7/26, pre-diluted; Ventana, Tucson, AZ, USA) and anti-CD3 (rabbit monoclonal clone 2GV6, pre-diluted; Ventana, Tucson, AZ, USA). Washings were performed using PBS/Tween20 pH 7.6 and reactions were detected using an HRP-conjugated compact polymer system HRP-DAB kit (UCS diagnostics, Rome, Italy). The presence of inflammatory infiltrate was evaluated as the percentage of CD45- or CD3-positive lymphocytes. The evaluations were carried out separately for each sample, using a double-blind method. In cases of divergent scoring, a third observer (ET) decided the final score.

### 2.4. Statistical Analysis

Analysis of data was performed using a Kruskal–Wallis test. When differences were found to be significant, analysis between the unmatched groups was elucidated with Dunn’s multiple comparison post hoc test. The significance level was set to 5% (SPSS ver. 16.01 for Windows—SPSS Inc., Chicago, IL, USA).

### 2.5. Toxicological Analysis

All chemicals and reagents used were of analytical grade. Potassium phosphate (monobasic and dibasic), methanol, hydrochloric acid, dichlorometane, isopropanol, ammonium hydroxide and N,O-bis(trimetilsilil)trifluoroacetamide+1%trimethylchlorosilane (BSTFA + 1%TMCS) were purchased from Sigma-Aldrich (Milan, Italy). The certified reference materials of cocaine, benzoylecgonine and their deuterated analogs were purchased from Lipomed AG (Fabrikmattenweg 4, CH-4144 Arlesheim, Switzerland) and Cerilliant (Round Rock, TX, USA). A solid phase extraction (SPE), performed with Strata™-X drug B (Phenomenex, 411 Madrid Avenue, Torrance, CA, USA), was applied to the analysis of cocaine and its main inactive metabolite, benzoylecgonine (BZE), in post-mortem femoral blood. Following extraction, cocaine and benzoylecgonine were identified and quantitated by gas chromatography coupled to mass spectrometry (GC-MS).

#### GC-MS Analysis

After the extraction procedure, the samples were injected into an Agilent 7890A GC system equipped with an Agilent 7683B series autosampler and interfaced with a single Agilent 5975C quadrupole mass spectrometer (Agilent Technologies, Palo Alto, CA, USA). The column was a 30 m long HP-5MS Agilent fused silica capillary column, 0.25 mm i.d. and 0.25 μm film thickness (Agilent Technologies, Palo Alto, CA, USA). The constant flow of the carrier gas (He) was 1 mL/min. A splitless injection mode was used.

The ionization mode was an electronic impact at 70 eV, and the detector voltage was 0.9 kV. The injector temperature was 270 °C. The initial oven temperature was 130 °C for 1 min, then increased at 25 °C/min to 300 °C, and held for five minutes. The transfer line temperature was maintained at 280 °C. The total time of analysis was 15 min.

A total of 1 μL of each sample was injected into the GC-MS in SIM mode. The ions of *m*/*z* 303 and 361 were, respectively, selected for cocaine and benzoylecgonine quantitative analysis.

## 3. Results

### 3.1. Morphological Analysis

The average heart weight was 380 g in cases and 365 g in controls.

The histological evaluations performed using HE, Masson’s trichrome, Sirius’ red and Van Gieson-stained sections are reported in Table 1.

Specifically, morphological cardiac lesions were detected in 86.7% of samples from the cocaine-positive group. Of note, only 20.0% of samples collected from the control group showed relevant morphological alterations. The cardiac lesions comprise the presence of focal wavy fibers, interstitial edema, fibrosis and myocardiosclerosis. Concerning the focal wavy fibers, in the control group they were detected in only 5 out of 30 cases (16.7%), whereas this alteration was higher in the cocaine-positive group, with 18 out of 30 cases (60.0%) showing the presence of wavy fibers. Among them, seven cases also had interstitial edema and congestion. Moreover, the cocaine-positive group showed 3 of 30 subjects (10.0%) with subendocardial fibrosis and 8 out of 30 patients (26.0%) with hemorrhagic extravasation. No interstitial edema, congestion, subendocardial fibrosis or hemorrhagic extravasation were observed in samples from the control group. Lastly, only two patients (6.7%) in the control group had myocardiosclerosis.

### 3.2. Immunohistochemical Analysis

The analysis conducted by immunohistochemical analysis revealed different percentages of positivity for the CD45 and CD3 biomarkers within the two examined groups (Table 2).

**Figure 2:**
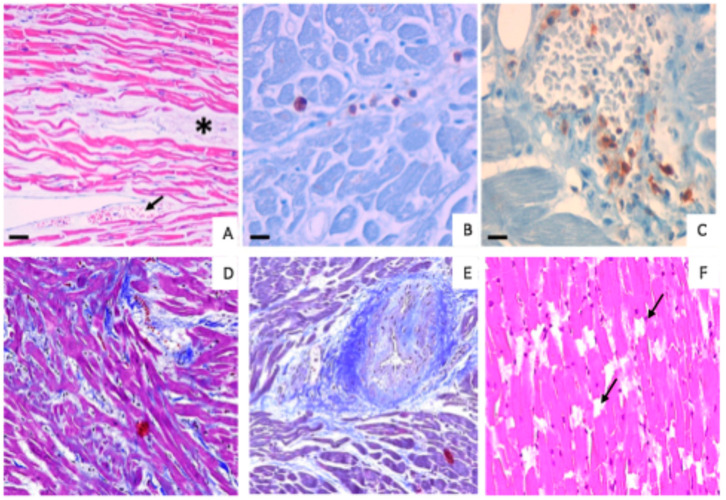
Morphological and immunohistochemical evaluation of cocaine-induced cardiac alterations. (**A**) HE image shows cardiac tissue of a cocaine-death-related subject with wavy fibers, edema (asterisk) and hemorrhagic extravasation (arrow). Scale bar represents 50 µm. (**B**) Few CD45-positive cells in the cardiac tissue of a traumatic-death-related subject. Scale bar represents 30 µm. (**C**) Numerous CD45-positive cells in the cardiac tissue of a cocaine-death-related subject. Scale bar represents 30 µm. (**D**) Masson’s trichrome where myocardial disarray is observed. (**E**) Masson’s trichrome: presence of medial–intimal hyperplasia of the microcirculation. (**F**) HE myofiber break-up (arrows).

In the control group, low percentages of positivity were found for both CD3 (3 out of 30 cases, 10%) and CD45 (6 of 30 cases, 20%). On the contrary, in subjects who died after cocaine intake, both immunological markers were more highly expressed; CD3 was found in 60% of cases and CD45 in 90% of cases, comprising 18 out of 30 cases for CD3 and 27 out of 30 cases for CD45, respectively (Figure 2).

### 3.3. Toxicological Results

Toxicological tests on the case sample showed a mean cocaine value of 3.23 mg/L, with a range of 0.46 mg/L–47 mg/L. Concerning cocaine metabolites, benzoylecgonine was detected with a mean concentration of 3 mg/L (Table 3). In the control group, toxicology was negative for alcohol and drugs.

## 4. Discussion

The pathophysiological pathways determining organic damage in cocaine users have been extensively studied but not yet fully comprehended in their complexity. Cocaine is known to exert adverse systemic effects following both acute intoxication and chronic use. One of the most affected organs is the heart, on which cocaine causes tissue alterations, directly involving both myocardial tissue and vascular structures. From a diagnostic perspective, it appears to be of the utmost importance to carry out, in addition to toxicological investigations, histopathological investigations in cases of suspected death related to cocaine intoxication, aimed at identifying the organic changes induced directly or indirectly by the intake of the drug and their influence on the causal determinism that contributed to death [35,36]. According to some authors, isolated cocaine levels cannot be used to explain the cause of death. In fact, the presence of low levels of cocaine only demonstrates cocaine use, though if the appropriate anatomical or histological changes are present, cocaine may be the cause of death even if it is not detectable in the blood [37].

To support the hypothesis of chronic drug use, keratin matrix analysis can be performed, which provides important information on consumption patterns or trends in intake. Hair analysis, in particular, allows the time window of substance detection to be expanded from months to years, depending on the length of the sample and considering the average hair growth of about 1 cm per month [38].

The importance of using histopathological methods in establishing the diagnosis in cases of acute intoxication by narcotics or drugs is also emphasized by several guidelines concerning the performance of judicial autopsies [39].

Based on these considerations, in the current study, we have evaluated histopathological changes induced by cocaine abuse in post-mortem cardiac samples. From a morphological point of view, the pathophysiology of cardiac alterations induced by cocaine has been well described [40]. In general, hearts of cocaine users are enlarged and in acute death, contraction bands can be observed. Another morphological alteration that can be observed in cocaine users is a form of “accelerated atherosclerosis”, especially involving young subjects [41].

Specifically, we found cocaine-related morphological alterations such as wavy fibers, edema, congestion, fibrosis and hemorrhagic extravasation. Interestingly, some of these changes (edema, hemorrhagic extravasation and subendocardial fibrosis) were only observed in the tissue samples from the study group and never in the control samples, suggesting a certain degree of specificity of these findings with respect to death from acute cocaine intoxication.

These findings are in line with previous studies that have highlighted that chronic cocaine use is associated with left ventricular hypertrophy, myocardial disarray, mononuclear cellular infiltration, perivascular fibrosis and collagen deposition [42]. The finding of myocardial fibrosis appears to be of particular relevance with regard to the determinism of death. Some authors [43] have in fact pointed out that the deposition of fibrotic tissue in particular sites such as the cardiac conduction system in chronic cocaine users could explain the onset of even fatal arrhythmias, and thus help clarify the determinism of death in cases of sudden death in which the only data are a history of cocaine use, a relatively frequent occurrence in forensic pathology.

Notably, immunohistochemical analysis showed higher levels of inflammatory cell infiltration in cases of cocaine-related deaths compared to the control group myocardium, suggesting a role for the inflammation response in the pathophysiology of myocardial damage in cocaine intoxication. It has been shown that cocaine promotes an elevated immune system activation state accompanied by decreased basal anti-inflammatory markers and increased pro-inflammatory mediators (such as TNF-α and IL1 β), which can lead to vascular and tissue damage [44].

Within cardiomyocytes, cocaine can affect cytoskeletal and mitochondrial structures and functions. Findings from in vitro data indicate that high doses of cocaine (50–100 mg/mL) have a severe effect on H9c2 cardiomyocytes’ viability and proliferation, whereas low doses (5–10 mg/mL) induce alterations in membrane stability [45]. Moreover, cocaine reduces the compaction among cells and promotes the loss of cellular architecture, suggesting possible implications for cardiotoxicity relating to hypertrophy and fibrogenesis. In adult rat isolated cardiomyocytes exposed to cocaine, in concentrations commonly used in chronic cocaine addiction, calcium currents across the sarcolemma membrane were increased, with a subsequent activation of calcium/calmodulin kinase II [46]. In turn, the increasing calcium release from the sarcoplasmic reticulum within cardiomyocytes led to cardiomyocyte hypertrophy and to cocaine-induced cardiac arrhythmias.

Another mechanism through which cocaine can impair heart structure and function is associated with oxidative stress processes. It is known that oxidative stress can impair several physiological mechanisms such as proliferation and cell death [47,48], especially in myocardial tissue, which, due to its high oxygen consumption, is particularly sensitive to damage caused by increased oxidative stress [49]. In a murine model, cocaine exposure is able to activate mechanisms related to oxidative stress in cardiomyocytes. A study on rats exposed to chronic cocaine administration (30 days) provides evidence of a decrease in glutathione (GSH) in hearts of cocaine-exposed rats, a sign of excessive reactive species and a slowed-down antioxidant system [50]. In mice, chronic administration of cocaine results in severe myocardial oxidative stress, along with activation of Nox2 oxidase and MAPK activation, responsible for myocyte damage [51]. In cocaine-administered rats (14 days), widespread contraction band necrosis (CBN), microfocal myocarditis and myocardial fibrosis were found, corresponding to a dysfunction of cardiac mitochondria under increased oxidative stress [52]. In addition, the authors registered an increased dephosphorylation of Connexin-43, a molecular modification associated with the degradation of the protein, which results in the weakening of intercellular communications and in the propagation of CBN. A retrospective human study, performed on endomyocardial biopsies of chronic cocaine users with dilated cardiomyopathy, showed a remarkable increase in myocyte expression of iNOS and nitrotyrosine, two markers of oxidative stress [53].

Similar results were obtained by other authors, whose findings showed a significant increase of iNOS, NOX2 and nitrotyrosine expression in subjects who died from cocaine acute intoxication. Regarding the dramatic increase of oxidative stress in acute cocaine deaths, the same study showed an elevation of expression in myocardial tissue of another oxidative stress marker, 8-OHdG [54]. These results underline that oxidative stress is a major mechanism of myocardial damage both in acute and chronic cocaine users.

The immunohistochemical analysis for common CD45 and CD3 showed a great increase in the group of cocaine users compared with the control group, in which the presence of CD45- and CD3-positive cells was very low, confirming the role of the inflammatory response in myocardial damage mechanisms.

The combination of CD45 and CD3 markers allows for a more comprehensive detection of the inflammatory infiltrate. Indeed, CD45 is a pan-leukocyte marker, while CD3 focuses on T cells. These markers are among those employed for phenotyping inflammatory infiltrates on endomyocardial biopsies after myocarditis [55,56]. The use of CD45 allows for the detection of a broad spectrum of immune cells, which can help identify any inflammatory response involving leukocytes in the heart tissue [57]. These cells may contribute to both acute and chronic inflammation, involved in myocarditis, ischemic injury, and chronic inflammatory heart diseases [58,59]. In addition, CD3 is specific for T lymphocytes and helps identify T cell-mediated inflammation. T cells, particularly in autoimmune myocarditis, contribute to tissue damage and the progression of chronic inflammation [60].

Myocardial inflammation is a characteristic feature associated with the use of substances of abuse. Despite limited research focused on quantifying inflammatory cells within the hearts of cocaine users [61], our discovery of elevated inflammation levels in the tested group aligns with other findings. Our study suggests that cocaine promotes a potentiation in the genesis of inflammation following both acute and chronic intake. Indeed, it has been demonstrated that cocaine can induce an inflammatory state. An in vitro study examined the effects of cocaine on cultured human peripheral blood T lymphocytes [62]. According to the study, cocaine promotes the stimulation of the CD3 receptor and the release of IL2 at the drug concentrations observed in the blood of a cocaine user. Clinical evidence also suggests that systemic inflammation is boosted by cocaine intake. Indeed, by analyzing blood samples and clinical data in a cohort of 50 women with cocaine-use disorder in rehab, compared with 19 control women, high amounts of both pro-inflammatory and anti-inflammatory cytokines have been found [63]. Other investigations on the subject have demonstrated that cocaine interacts with dopamine receptors expressed on certain types of immune cells, such as T lymphocytes, B lymphocytes and NK cells. These receptors are involved in various cellular processes such as differentiation and activation, underlining the complexity of the substance’s interactions at the cellular level [64].

Moreover, according to some authors, regarding the systemic activation of the immune response, it has been highlighted that the concomitant use of cannabis and cocaine can lead to higher systemic levels of lipopolysaccharide (LPS), C-reactive protein and IL-6 [65].

Interestingly, the Il-6 cytokine is closely related to the severity of symptoms of cocaine withdrawal. The correlation between high levels of IL-6 in blood and cocaine’s health impairment has been determined at the neurological level, where it strongly reduced mental executive function in crack cocaine-dependent individuals [66]. Here, the activated inflammatory process could be associated to cocaine-related death processes. Indeed, chronic abuse of crack cocaine presents with increased brain-derived neurotrophic factor (BDNF), IL-1 and TNF-β levels compared to healthy controls, suggesting an activation of the immune and inflammatory systems as a compensatory mechanism to the neuronal death [67]. A post-mortem study on central nervous systems highlighted that acute lethal intoxication following chronic abuse of cocaine and/or heroin induces neuroinflammation in humans, especially in terms of leukocyte adhesion (CD3-positive cells) and extravasation of lymphocytes within the brain, which were absent in controls [68]. Such inflammatory characterization is missing in the cardiac region, although systemic inflammation could promote this effect on the heart. Concerning the systemic inflammation response, it has been shown that this process can also involve the kidneys. A study found a high incidence of glomerular and periglomerular sclerosis associated with interstitial mononuclear cell infiltration in cocaine-related deaths [69].

Concerning our findings, must be clarified that, although minimal, the presence of inflammatory cells in subjects with no cocaine addiction has been detected, indicating that inflammation is a common process that occurs during ischemic events. This has been demonstrated in subjects who were affected by ischemic heart disease, in which a diffuse myocardial inflammation, consisting of an infiltrate of activated T lymphocytes, has been found [70]. These activated inflammatory cells could be related to myocardial infarction, because an antigenic stimulus present in the myocardium could stimulate an immune response that would be the critical event capable of precipitating arterial occlusion. However, in cocaine-positive subjects, the levels of inflammation were notably higher compared to the control group. It follows that the genesis of the inflammatory state, which could be responsible for myocardial infarction in ischemic heart disease, would be accelerated in drug-addicted subjects, and this would explain the high frequency with which drug-addicted subjects are affected by acute myocardial infarction.

Although the results obtained appear promising, it must be stressed that our study may have certain limitations. Firstly, there were a limited number of cases and control samples included in the analysis, and there were a lack of toxicological studies conducted on the hair matrix to determine the possible state of chronic toxicity.

## 5. Conclusions

The clinical implications of our findings are significant in the context of public health. In the era of personalized medicine [71,72], recognizing the specific cardiac alterations associated with cocaine use can aid healthcare professionals in early detection and intervention. In this scenario, our histological and immunohistochemical post-mortem analysis sheds light on the complex relationship between cocaine use and cardiac alterations. The findings reported here highlight the need for continued research to elucidate the underlying mechanisms and develop targeted therapeutic strategies to mitigate the adverse cardiac effects associated with cocaine use. The results obtained could also represent a useful tool at the disposal of forensic pathologists in defining, from a diagnostic point of view, deaths related to cocaine abuse, thus emphasizing the importance of an integrated analysis between circumstantial, toxicological and autopsy data. In order to improve the diagnostic procedure in these cases, it seems advisable to take into account the availability of operational reference protocols, which, although they cannot be defined as guidelines due to the methodology with which they are elaborated, represent an authoritative reference in the field [73,74].

## Figures and Tables

**Table 1:** Morphological alterations in cardiac samples.

	Traumatic Deaths (%)	Cocaine-Related Deaths (%)
Morphological cardiac lesions	86.7	20.0
Wavy fibers	60.0	16.7
Subendocardial fibrosis	0	10.0
Hemorrhagic extravasation	0	26.0
Interstitial edema and congestion Myocardial disarray Myofiber break-up Intimal medial hyperplasia of the microcirculation	0	23.3

**Table 2:** Percentage of subjects with CD3 or CD45 inflammatory infiltrate.

	Traumatic Deaths	Cocaine-Related Deaths
CD-3 positive cells	10%	60%
CD-45 positive cells	20%	90%

**Table 3:** Toxicological results for each case in the study group.

Case No.	Sex/Age	Toxicology/Femoral Blood (Cocaine)	Toxicology/Femoral Blood (Benzoylecgonine)
1	Male/38	0.47 mg/L	1.10 mg/L
2	Male/43	0.50 mg/L	1.30 mg/L
3	Male/26	1 mg/L	2 mg/L
4	Female/35	1.30 mg/L	2.30 mg/L
5	Male/20	0.88 mg/L	1.40 mg/L
6	Male/47	0.46 mg/L	1.50 mg/L
7	Male/29	1.50 mg/L	3 mg/L
8	Female/31	14 mg/L	11 mg/L
9	Female/39	0.63 mg/L	2 mg/L
10	Male/55	1.20 mg/L	2.50 mg/L
11	Male/34	0.90 mg/L	1.70 mg/L
12	Male/28	1.20 mg/L	2.80 mg/L
13	Female/40	0.85 mg/L	1.60 mg/L
14	Male/21	1.80 mg/L	3.20 mg/L
15	Male/37	1.40 mg/L	2.60 mg/L
16	Male/23	47 mg/L	7.48 mg/L
17	Male/24	0.75 mg/L	1.70 mg/L
18	Male/46	0.94 mg/L	2 mg/L
19	Male/22	1.30 mg/L	1.90 mg/L
20	Male/19	1.50 mg/L	3 mg/L
21	Male/23	0.80 mg/L	2.80 mg/L
22	Male/33	2 mg/L	3.30 mg/L
23	Male/28	0.90 mg/L	1.60 mg/L
24	Male/24	1.20 mg/L	2 mg/L
25	Male/34	0.70 mg/L	1.40 mg/L
26	Male/30	0.17 mg/L	2.43 mg/L
27	Male/48	2.20 mg/L	3.50 mg/L
28	Male/21	5.22 mg/L	5.12 mg/L
29	Male/42	0.80 mg/L	1.50 mg/L
30	Male/57	1.30 mg/L	10.20 mg/L

## Data Availability

The data used to support the findings of this study are available on request from the corresponding author.
